# Scope of centres of expertise for rare diseases in European countries where they exist

**DOI:** 10.1186/1750-1172-7-S2-A5

**Published:** 2012-11-22

**Authors:** Charlotte Rodwell, Ségolène Aymé, Kate Bushby

**Affiliations:** 1 European Union Committee of Experts on Rare Diseases (EUCERD) Scientific Secretariat, INSERM US 14, Paris, France; 2 Network of Excellence TREAT-NMD, Institute of Human Genetics, Newcastle University, Newcastle, UK

## 

The development of centres of expertise (CE) and European Reference Networks (ERN) in the field of rare diseases (RD) is encouraged in the Council Recommendation on an Action in the Field of RD (2009/C 151/02) (8 June 2009) and most recently in the Directive on the application of patients’ rights in cross-border healthcare (2011/24/EU) (9 March 2011) as a means of organising care for thousands of heterogeneous RD affecting scattered patient populations across Europe. The European Union Committee of Experts on Rare Diseases (EUCERD http://www.eucerd.eu) issued a set of *Recommendations on Quality Criteria for Centres of Expertise for Rare Diseases in Member States *[1] to guide Member States (MS) in this area.

The scope of CE in terms of disease coverage is an important issue as the expectation is to provide CE for all RD patients’ needs at national level. Denmark, France, Norway, Spain and the UK, for example, have already identified existing expertise at national level and/or established centres specialised in some RD/groups of RD which have proven to be very efficient in improving quality of care. The Scientific Secretariat of the EUCERD examined the current scope of CE in countries where they exist. CE were grouped by medical area. Three or more MS have designated centres for: juvenile arthritis/ paediatric rheumatological diseases, developmental anomalies and malformations/dysmorphology, hereditary cardiac diseases, dermatological diseases, epidermolysis bullosa, pituitary diseases or hypothalamic-hypophyseal diseases, lysosomal diseases, Prader-Willi syndrome, Fabry disease, mitochondrial diseases, haemophilia/ constitutional bleeding disorders, mastocytosis, hereditary diseases of the metabolism, porphyrias, epilepsies, neuromuscular diseases, amyotrophic lateral sclerosis, pulmonary diseases, severe pulmonary hypertension, cystic fibrosis, hereditary immune deficiencies, opthalmological diseases, genetic kidney disease, cranofacial anomalies, neurofibromatosis, Rendu-Osler disease.

On the basis of this experience, a consensus can be thus identified that centres are required for around 12 groups of RD, 30 subgroups, and 26 individual diseases where centres currently exist in two or more countries. Most of these groups of RD fit into the traditional organisation of healthcare by medical area. However some grouping outside of traditional medical specialities is necessary, e.g. diseases of connective tissue, rare bone diseases, neurofibromatosis, multimalformation syndromes with intellectual disability, mitochondrial diseases, lysosomal diseases, any multi-systemic complex disease, etc. This analysis could be of use for MS currently considering the organisation of CE for RD.

This work was carried out by the EUCERD Scientific Secretariat with the support of EC Joint Action N°20082291.
